# A Modular and Affordable Time-Lapse Imaging and Incubation System Based on 3D-Printed Parts, a Smartphone, and Off-The-Shelf Electronics

**DOI:** 10.1371/journal.pone.0167583

**Published:** 2016-12-21

**Authors:** Rodrigo Hernández Vera, Emil Schwan, Nikos Fatsis-Kavalopoulos, Johan Kreuger

**Affiliations:** 1 Department of Medical Cell Biology, Uppsala University, Uppsala, Sweden; 2 Gradientech AB, Uppsala Science Park, Uppsala, Sweden; Pohang University of Science and Technology, REPUBLIC OF KOREA

## Abstract

Time-lapse imaging is a powerful tool for studying cellular dynamics and cell behavior over long periods of time to acquire detailed functional information. However, commercially available time-lapse imaging systems are expensive and this has limited a broader implementation of this technique in low-resource environments. Further, the availability of time-lapse imaging systems often present workflow bottlenecks in well-funded institutions. To address these limitations we have designed a modular and affordable time-lapse imaging and incubation system (ATLIS). The ATLIS enables the transformation of simple inverted microscopes into live cell imaging systems using custom-designed 3D-printed parts, a smartphone, and off-the-shelf electronic components. We demonstrate that the ATLIS provides stable environmental conditions to support normal cell behavior during live imaging experiments in both traditional and evaporation-sensitive microfluidic cell culture systems. Thus, the system presented here has the potential to increase the accessibility of time-lapse microscopy of living cells for the wider research community.

## Introduction

3D printing was developed in the 1980s [1] but it was not until recently that affordable desktop printers became commercially available. Lately, the dissemination of 3D printing has been remarkable and the sales of desktop 3D printers costing less than 5,000 USD increased by 69.7% in 2015 to reach a total of 278,385 units sold worldwide [2]. The greater availability of 3D printers will arguably lower the threshold for researchers in the life sciences to produce their own custom-made research tools. The do-it-yourself manufacturing revolution has the potential to bring some research technologies that were previously out of reach due to high equipment costs into low-resource environments, including laboratories in developing countries and schools. Indeed, over the past years, several groups worldwide have started to develop do-it-yourself research tools such as micropipettes, micromanipulators, syringe pumps, and webcam-based microscopes [3–5].

Microscopy is a central technique in biomedical research. In particular, time-lapse imaging is useful as it allows for the study of cell dynamics both in vitro an in vivo. However, live cell imaging is one of the areas where high prices of commercially available systems have restricted this methodology mostly to well-funded research institutions. One of the main reasons behind the high prices of live imaging systems is the need for strict environmental control to guarantee normal cell behavior during the imaging period. Thus, additional expensive equipment is required to maintain stable and optimal temperature and pH conditions for cell growth, to minimize exposure to light to reduce phototoxicity, and to minimize evaporation to avoid changes in osmolarity [6, 7].

Here, we describe an affordable time-lapse imaging and incubation system (ATLIS), which is modular in design and enables the transformation of simple inverted microscopes into live imaging systems for less than 300 USD. The ATLIS was assembled from a set of custom-designed 3D-printed parts, a smartphone, and off-the-shelf electronic components. We provide complete information on how to assemble the system as well as data to demonstrate that the ATLIS provides the adequate environmental conditions to support normal cell proliferation and behavior during time-lapse imaging experiments of regular cell cultures. Further, the addition of a humidifying module was shown to make the ATLIS compatible with imaging of cell culture systems that are highly sensitive to evaporation.

## Results and Discussion

### System overview

**Fig 1:**
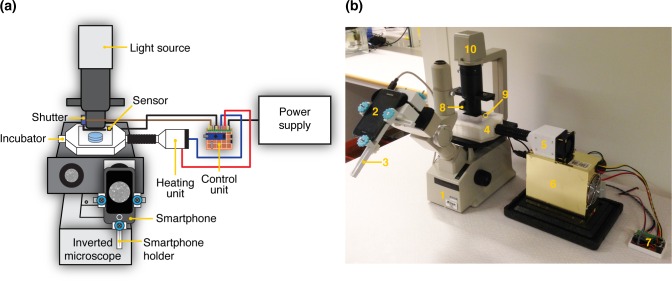
ATLIS: an affordable system for time-lapse imaging and incubation of cells. **(a)** The basic version of ATLIS was assembled by the combination of four modules. The *imaging module*, composed of a smartphone, a 3D-printed holder, and a motorized shutter, was designed to capture images of cells at a fixed interval minimizing their exposure to light. The *heating unit* was created to warm up the air introduced into a 3D-printed *onstage incubator* where cells are kept. Finally, the *control unit* was programmed to maintain a constant temperature inside the incubator by adjusting the power supplied to the heating unit. **(b)** A photograph of the ATLIS mounted on a Nikon TMS microscope fitted with a Samsung Galaxy S I9000 smartphone. (1) Nikon TMS microscope; (2) Samsung Galaxy S I9000 smartphone; (3) smartphone holder; (4) onstage incubator; (5) heating unit; (6) power supply; (7) control unit; (8) shutter; (9) temperature sensor; (10) light source.

The ATLIS described here was designed to enable the transformation of simple inverted microscopes, regardless of brand or model, into live cell imaging systems at a fraction of the cost of currently available commercial solutions. The ATLIS was built using a set of custom-designed 3D-printed parts, off-the-shelf electronic components, a smartphone, and standard hardware. The system was designed to be modular (**Fig 1**) and can be divided into four main parts: an imaging module, a heating unit, an onstage incubator, and finally a control unit. The assembly and operation of each of these modules will be described in the following sections.

### Imaging module

**Fig 2:**
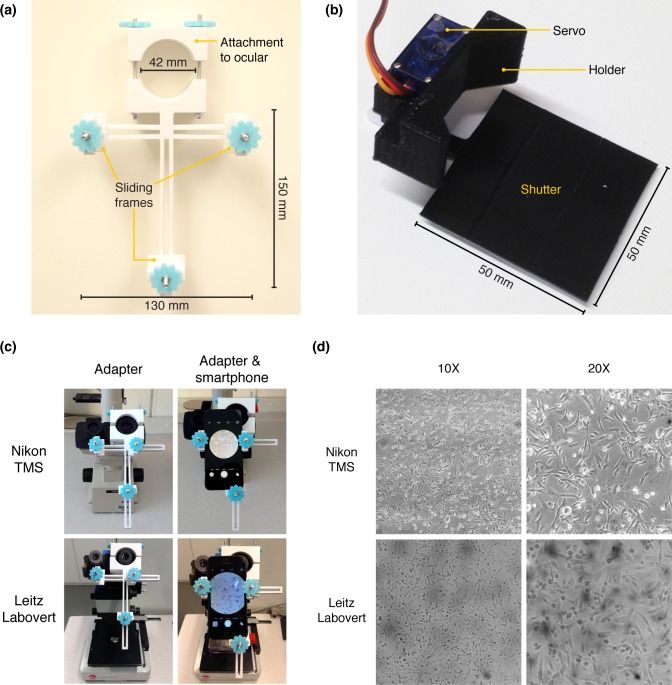
Imaging module. **(a)** The smartphone holder was designed to allow for stable fixation of most currently existing smartphones to microscopes having oculars of up to 42 mm in diameter. Sliding frames (that could be fixed by fastening the nut in each frame) in the holder facilitated the alignment of the smartphone’s camera to the ocular. **(b)** The shutter was built from a servomotor, a plastic shutter disc, and a 3D-printed connector. The servomotor was used to open or close the shutter in response to commands from the control unit. **(c)** Examples of two different microscopes fitted with the smartphone holder and an iPhone 5. **(d)** Images of MDA-MB-231 breast cancer cells captured with an iPhone 5 and the smartphone holder using the 10X- and 20X-objectives of each indicated microscope.

The imaging module was designed to capture high quality images at a fixed interval using the camera of a smartphone while at the same time minimizing the exposure of cells to light. This module was assembled from a 3D-printed custom-made smartphone holder, a motorized shutter, and a smartphone. The holder (**Fig 2A**) was used to attach the smartphone to one of the microscope’s oculars as well as to adjust and stably fix its position in order to capture high-quality images throughout the duration of the experiment. The holder was based on a design originally deposited at Thingiverse (http://www.thingiverse.com/thing:431168) that was modified to make it compatible with most commonly available smartphones and with microscopes having oculars of up to 42 mm in diameter.

The shutter (**Fig 2B**) was made from a 3D-printed connector, a servomotor, and a shutter disc. The connector was designed to allow for stable fixation of the shutter to the microscope so that the bluetooth-controlled servomotor can move the shutter disc to block or let through light emitted from the microscope lamp. The shutter disc was made from a piece of polyethylene terephthalate cut to shape and covered with black tape. The imaging system was tested on a Nikon TMS microscope and a Leitz Labovert microscope (**Fig 2C**) to analyze its performance with different microscopes and images of human breast adenocarcinoma cells (MDA-MB-231) were captured (**Fig 2D**). A comparison of the images obtained with the two microscopes using the 10X objectives (**Fig 2D**) showed that the images acquired using the Nikon microscope were of a higher quality than those captured using the Leitz microscope (both objectives had identical specifications; see Materials and Methods). Thus, the microscope optics ultimately determined the quality of the images obtained. Further, in order to test the ability of the system to take high-magnification pictures, images of human embryonic kidney (HEK) cells with up to 400X magnification were captured using the imaging module coupled to the Nikon TMS microscope (**S1 Fig)**.

### Heating unit

**Fig 3:**
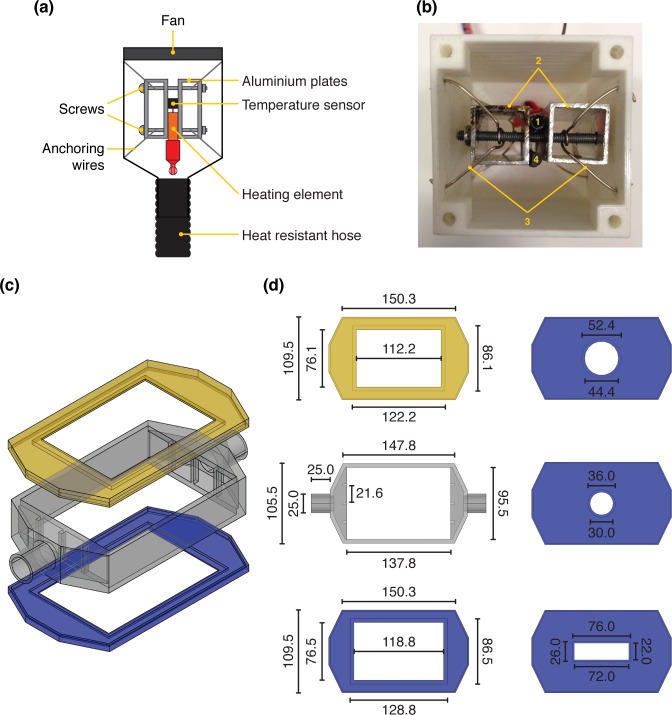
Heating unit and incubation module. **(a)** The heating unit was constructed using a fan, a custom-built heating core, and a 3D-printed case designed to connect to the incubator using a heat-resistant hose. As a safety measure, a temperature sensor was placed close to the heating core to monitor its temperature so that the heating could be turned off when the temperature exceeded a preset safety limit. **(b)** Photograph of the heating unit where the fan has been removed to show the position of the heating element (1), aluminum plates (2), the anchoring wires (3), and the limiting temperature sensor (4). **(c)** Exploded view of the onstage incubator. The 3D-printed incubator was built from three parts: a lid (yellow), a main body (gray), and a bottom plate (blue). Four different bottom plates were designed to fit the most common types of cell culture ware. **(d)** Main dimensions (in mm) of the incubator lid and main body as well as for the different bottom plates designed to fit a multi-well plate, a 60 mm Petri dish, a 35 mm Petri dish or a microscope slide, respectively.

The heating unit (**Fig 3A and 3B**) was designed to warm up the air that flows into the incubator in order to maintain cells at an optimal temperature (*e*.*g*. 37.0°C). The core of the heating unit was built using a heating element placed between two commercial-grade square aluminum profiles (20 x 20 x 50 mm each) kept together with two 50 mm long M4 screws. This core piece was placed inside a 3D-printed case and kept centered and suspended in the air using 4 anchoring wires to avoid contact with the plastic walls. A 60 mm fan was affixed to the back of the case, and the air outlet on the opposite end connected to the inlet of the incubation chamber using a heat-resistant hose. Due to the low glass transition temperature of many of the thermoplastics used in fused deposition modeling printers, monitoring the temperature inside the heating unit was important. To this end, we placed a temperature sensor (Dallas DS18B20) close to the heating element to monitor the temperature of the heating core and added a temperature control failsafe to disable heating if the temperature exceeds a preset safety limit.

### Onstage incubator

The onstage incubator was designed to grow and maintain cells at a stable temperature in cell culture dishes, multi-well plates, or other cell culture systems compatible with imaging. The incubator was designed as a three-piece system with a lid, a body, and a bottom plate (**Fig 3C and 3D**). The lid was designed with a central opening where a 12.0 by 8.0 cm piece of transparent plexiglas (polymethyl methacrylate) was glued. This opening was created so that all parts of a multi-well plate can be exposed to light and imaged. The main body was designed to have an inlet for warm air coming from the heating unit, a central section, and an air outlet. Three columns evenly distributed at each end of the central section were included to promote air mixing. Finally, four different interchangeable bottom plates (**Fig 3D**) were designed to accommodate different types of cell culture ware, i.e. microtiter plates, 35 and 60 mm culture dishes, as well as microscope-slide sized cell culture chambers (including microfluidic systems).

### Control unit

**Fig 4:**
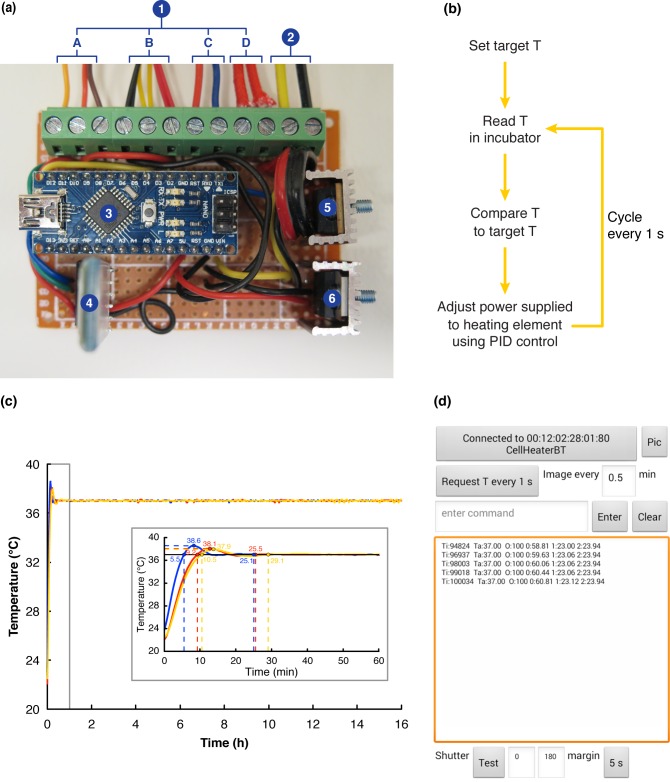
The control unit and the ATLIS Control app. **(a)** Photograph of the ATLIS control unit. (1) The power outlets for the shutter’s servomotor (A), the temperature sensors (B), the fan (C), and the heating element (D) are indicated. (2) Power supply to the control unit. (3) Arduino Nano 3.0 ATmega328 microcontroller. (4) Bluetooth communication module. (5) Power transistor for heating element control. (6) Voltage regulator. **(b)** Overview of the temperature control loop. The temperature inside the incubator was continuously monitored by a sensor and compared to the target temperature in order to adjust the power supplied to the heating element. **(c)** Temperature curves for three independent experiments showing the temperature inside the incubator during a 16 h period. Inset (gray box): magnification of the temperature behavior during the first hour of the three experiments. The time to reach 37.0°C, the time to temperature stability, as well as the maximum air temperature reached have been indicated for each experiment. **(d)** Screenshot of the ATLIS Control app designed to allow the user to set the main parameters for each experiment (e.g. target temperature and time interval between images) and to display temperature reads and the last picture taken.

The control unit (**Fig 4A**) was built using an Arduino 3.0 NanoAT328 microcontroller, a bluetooth communication module, a power transistor, and a voltage regulator. The control unit was connected to a standard computer power supply unit and then used to provide power at the appropriate voltages to the other components of system, *i*.*e*. the heating element (12 V), the fan (12 V), the temperature sensors (5 V) and finally the shutter’s servomotor (5 V). The control unit was used to operate the shutter’s servomotor via bluetooth, and also to control the temperature inside the incubator by adjusting the power supplied to the heating element.

### Temperature control

Achieving and maintaining an optimal and stable temperature throughout the duration of the experiment is vital when cell behavior is to be recorded by live imaging. In the ATLIS, precise temperature control was achieved using a PID controller operated according to the control loop shown in **Fig 4B**. A temperature sensor (Dallas DS18B20) was placed inside the incubator to send temperature reads to the Arduino microcontroller using the one-wire communication protocol at a sampling rate of 1 Hz. The microcontroller was programmed to compare the incubator’s temperature to the target temperature set by the user and based on this information (together with the preset K_p_, K_i_, and K_d_ values) adjust the power supplied to the heating element. The code for the microcontroller can be found in the **S1 Microcontroller Code**.

In an initial set of experiments the K_p_, K_i_, and K_d_ values were adjusted to obtain a fast response with a reasonable overshoot (< 2.0°C). The settings were finally optimized for this system configuration to 8.0, 0.5, and 0.1, respectively. In three independent experiments, the target temperature was reached in less than 11 min with only a moderate temperature overshoot. The maximum air temperature reached was 38.6°C and the time that the system’s air temperature was above 37.0 ± 0.1°C was less than 20 min for the three experiments. Moreover, in all cases the system reached steady state behavior (here defined as 37.0°C ± 0.1°C) in less than 30 min (**Fig 4C**).

### ATLIS Control app

In order to provide the user with an interphase to configure each experiment, we used MIT’s App Inventor to develop the ATLIS Control app (**Fig 4D**), compatible with Android operating smartphones and available free of charge (http://ai2.appinventor.mit.edu/#5955603006750720). The ATLIS Control app was designed to allow the user to set the target temperature and the imaging interval as well as other operating parameters (*i*.*e*. the controlling and limiting temperature sensors; the shutter’s positions; the K_p_, K_i_, and K_d_ values; and the interval at which temperature reads are stored). Additionally, the ATLIS Control app was programmed to allow monitoring of the ongoing experiment by displaying the recorded temperature reads and the last picture taken. Moreover, the app was also designed to enable the user to upload pictures and temperature logs in order to monitor the experiment remotely. A list of the commands needed to set the different parameters in the app can be found in the **S1 Table**.

### Live cell imaging using the ATLIS

**Fig 5:**
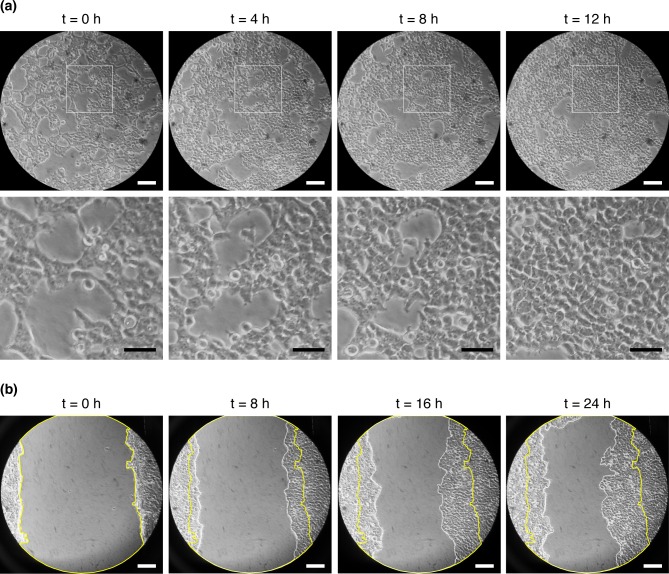
Time-lapse imaging using the ATLIS. **(a)** HEK cells grown in a 6-well plate were monitored using time-lapse imaging for 12 h. Scale bars: white 100 μm; black 50 μm. **(b)** Scratch-wound assay performed with HEK cells grown in a 6-well plate. Yellow lines delimit the original wound area while white lines show the collective cell migration front at each time point. Scale bars represent 100 μm. For both experiments the ATLIS was mounted on a Nikon TMS microscope using the 20X objective together with 10X magnification in the eyepiece, and images were captured using a Samsung Galaxy S I9000.

Human embryonic kidney (HEK) cells were analyzed in the ATLIS system in a 12 h long time-lapse microscopy experiment. As shown in **Fig 5A** and in the **S1 Video**, the ATLIS provided adequate environmental conditions for cells to proliferate normally. Indeed, no evidence of cell death was observed and cells preserved a normal phenotype throughout the duration of the experiment. Furthermore, a five-megapixel smartphone camera was found to be capable of producing high-resolution images comparable to those taken by microscope-dedicated cameras present in commercial live cell imaging systems (**Fig 5A**).

Next, an in vitro scratch wound assay was performed, as this is a commonly used assay to analyze cell polarization followed by collective cell migration [8, 9]. HEK cells were monitored by time-lapse imaging for 24 h using the ATLIS, and the cells were shown to migrate and proliferate efficiently to reduce the size of the scratch wound over time (**Fig 5B** and **S2 Video**). These long-term imaging experiments were performed using a HEPES-buffered cell culture medium to eliminate the need for CO_2_ administration, as HEPES, unlike bicarbonate/CO_2_ buffered media, can maintain physiological pH conditions independent of the atmospheric CO_2_ levels.

### Time-lapse imaging of cells grown in evaporation-sensitive systems

**Fig 6:**
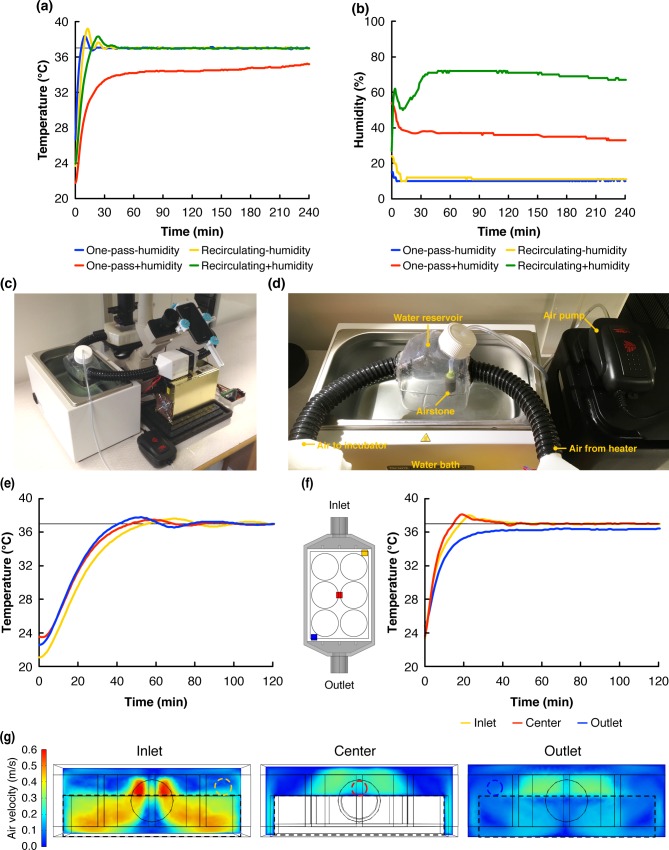
ATLIS setup with air humidification. **(a)** Without humidification the ATLIS reached and maintained the target temperature (37.0°C) in a one-pass (blue line) or a recirculating-loop configuration (yellow line). When humidification was added, the system was incapable of reaching the desired temperature in the one-pass configuration (red line). However, in a recirculating-loop configuration with humidification (green line) the system reached and maintained the target temperature. **(b)** Without humidification, the relative humidity inside the incubator stabilized around 10% regardless of system configuration. The humidifying module increased humidity up to 35% in the one-pass configuration and up to 70% when the ATLIS was operated in a recirculating-loop configuration. **(c)** Photograph of the ATLIS mounted on a Nikon TMS microscope in the recirculating-loop configuration with the humidifying module. **(d)** Photograph showing all the components of the humidifying module. **(e)** The temperature in the liquid inside a vessel placed in the incubator over time in three independent experiments using a recirculating loop together with the humidifying module. **(f)** Analysis of the temperature at different positions in the incubator in a recirculating-loop configuration with additional humidity. **(g)** Simulation of the airflow inside the incubator unit. Black dashed lines mark the edges of the multi-well plate; dashed circles indicate the positions of the temperature sensor.

Live imaging of cells cultured in microfluidic systems can be challenging as the small liquid volumes (μL to nL range) in such systems makes them sensitive to evaporation. Indeed changes in the concentration of proteins and salts in the cell culture media may result in altered cell behavior, or even lead to cell death. Here, cells were grown in a PDMS-based microfluidic chip holding a total volume of ~12 μL. Indeed, the high gas permeability of PDMS together with the flow of warm air in the ATLIS resulted in a rapid drying out of the media-filled channels where the cells were grown. The relative humidity inside the incubator of the ATLIS was measured using a digital hygrometer, and shown to stabilize around 10%, reflecting a dry environment (**Fig 6B**). Next, the system was operated using a recirculating air loop (**Fig 6C**) where warm air coming out from the incubator was returned to the heating unit. However, this did not solve the problem of evaporation of water from the microfluidic system and, to this end, a humidifying module was developed and added to the system.

The humidifying module was made using a water reservoir where an inlet was created to introduce the warm air coming from the heating unit and an outlet through which the warm and humidified air was led to the incubator (**Fig 6D**). To increase water transfer to the air, an airstone connected to an air pump was introduced. In addition, the water reservoir was placed inside a standard water bath set at 45°C. Using the humidifying module in a one-pass configuration increased humidity up to 35% but the system failed to reach the desired temperature, suggesting that the heating power of the ATLIS was insufficient to heat the humid air to the desired temperature. However, when the system was operated in a recirculating-loop configuration, the system reached the desired temperature and the humidity inside the incubator was shown to stabilize around 70% (**Fig 6A and Fig 6B**). Importantly, the introduction of the humidifying module did not affect the air temperature behavior greatly, as the system was shown to reach 37.0°C after 17.0 min and steady state behavior around 43.0 min (**Fig 6A**).

Analysis of the liquid temperature within a vessel placed inside the incubator using a recirculating loop with humidity (**Fig 6E**) showed that the liquid reached 37.0°C after ~50 min and that the temperature stabilized after ~100 min. Importantly, these experiments also showed that the temperature overshoot observed in the liquid was smaller than that observed in the air, as the maximum temperature recorded throughout the three experiments was 37.8°C.

Analysis of the temperature in different positions within the incubator (**Fig 6F**) showed no differences between the central region and the inlet region. However, the temperature was shown to reach 37.0°C after 209.0 min in the corners close to the outlet of the incubator. Simulation of the airflow inside the incubator with a multi-well plate inside to mimick the experimental situation revealed that there indeed was little airflow in the top corners of the outlet region, providing an explanation for the additional time needed to heat the air in this region compared to the rest of the incubator (**Fig 6G** and **S2 Fig)**.

**Fig 7:**
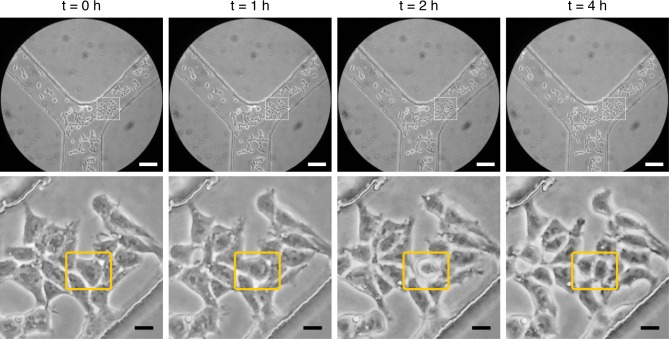
Imaging of HEK cells grown in microfluidic channels. HEK cells were imaged for 4 h with a Samsung Galaxy S I9000 using the ATLIS with the humidifying module (recirculating-loop configuration) mounted on a Nikon TMS microscope with 10X magnification in the eyepiece and using a 20X objective. No signs of evaporation were detected in the microfluidic system and the cells moved and proliferated normally. White boxes delineate magnified areas shown in the bottom row. A proliferating cell can bee seen in the area marked by a *yellow box*. The lower channel was 250 μm across, while the narrower channels at the top were 125 μm wide. All channels were 100 μm in height. Scale bars: white 100 μm; black 20 μm.

Finally, HEK cells were seeded in a microfluidic cell culture system and imaged for 4 h using the ATLIS in a recirculating-loop configuration with humidity. This configuration was shown to provide enough humidity to prevent measurable evaporation and thus to allow for time-lapse imaging of cells grown in highly evaporation-sensitive microfluidic channels for 4 h in ~12 μL of cell medium under no-flow conditions (**Fig 7** and **S3 Video**).

### Cost of the ATLIS components

The system developed in this study makes it possible to transform any simple inverted microscope into a time-lapse imaging system. A detailed list of all the system’s components, together with links to the retailers (when available) and prices paid are shown in the **S2 Table**. For all 3D-printed parts, the prices shown represent an average based on offers from three independent 3D-printing services. The total cost of all the components (excluding the smartphone and the microscope) used to build the ATLIS was 277 USD (including the humidifying module). This represents a drastic reduction in price compared to commercially available systems. Importantly, the price of the ATLIS can be further reduced, as several electronic components that in the present study were bought in regular retail stores also can be found online at lower prices (sites providing free international shipping for all the components bought in retail stores are provided in the **S2 Table**). Furthermore, the price of the ATLIS can also be greatly reduced for anyone with access to a 3D printer as the 3D-printed parts account for ~50% of the system’s total price.

## Conclusions

This study describes a modular system made from custom-designed 3D-printed parts, a smartphone, and off-the-shelf electronic components that allows for the transformation of simple inverted microscopes into time-lapse imaging systems for less than 300 USD. The system, called ATLIS, was used to successfully carry out time-lapse imaging of traditional cell cultures as well as of cells grown in highly evaporation-sensitive microfluidic systems. The system presented here has the potential to make time-lapse imaging of living cells more accessible to the wider research community.

## Materials and Methods

### 3D-printing

All parts were printed in polylactic acid with a RepRap Prusa printer equipped with a 0.5 mm nozzle using 0.3 mm layers. STL files for all parts are available upon request.

### Temperature and humidity determination

For all temperature and humidity monitoring experiments the multi-well plate bottom of the incubator was used and a 6-well plate placed inside the incubator. The temperature was recorded at 1 min intervals using a Dallas DS18B20 sensor. Relative humidity was measured at 1 min intervals using a digital hygrometer (Exo Terra PT2477) placed in the central region of the incubator.

### Cell culture

MDA-MB-231 and HEK cells were routinely cultured in Dulbecco’s Modified Eagle Medium (DMEM; Gibco 319966021) supplemented with 10.0% fetal bovine serum (FBS; Gibco 10270106) at 37.0°C in a humidified atmosphere with 5.0% CO_2_.

### Imaging

Images of MDA-MB-231 cells were taken with an iPhone 5 attached to the ocular using the above described smartphone holder coupled to a Nikon TMS microscope using the 10X (Nikon E10/0.25 160/- Ph1 DL) and 20X (Nikon 20/0.4 160/1.2 Ph2 DL) objectives and to a Leitz Labovert microscope using the 10X (Leitz EF 10/0.25 160/- PHACO1) and 20X (Leitz EF L20/0.32 160/- PHACO1) objectives. For time-lapse imaging experiments, HEK cells were seeded in 6-well plates or microfluidic chips and allowed to attach overnight. Before imaging the medium was changed to DMEM supplemented with 25 mM HEPES (Gibco 21063–029) and 10.0% FBS. For all time-lapse imaging experiments the ATLIS was mounted on the Nikon TMS microscope and pictures were taken with a Samsung Galaxy S I9000 using the 20X objective.

### Simulation

The simulation of the airflow inside the incubator in the presence of a multi-well plate was performed using Comsol Multiphysics 5.1 assuming: an airtight assembly, an incoming air temperature of 37.0°C, and an airflow of 30.5 m^3^/h.

### Fabrication of microfluidic chips

Microfluidic chips were produced in PDMS (Sylgard 184, Dow Corning) by soft lithography [10]. Inlets and outlets were punched using a 1 mm biopsy punch and the PDMS chips were bonded to glass microscope slides after plasma treatment. Prior to cell seeding, the system was primed with ethanol, washed extensively with PBS and coated with 0,005% poly-L-lysine (Sigma P4707) for 30 min to allow for efficient attachment of cells. Cells were routinely seeded at a concentration of 5x10^6^ cells per mL.

## Supporting Information

S1 Fig High-resolution images of HEK cells. HEK cells were imaged using the imaging module of the ATLIS mounted on a Nikon TMS microscope with 10X magnification in the eyepiece using 10X, 20X and 40X objectives.
(TIF)

S2 Fig 3D model showing the sensor positions and simulation planes. **(a)** Scheme showing the position of the sensor in each experiment. The multi-well plate placed inside the incubator in the simulation is depicted in dark grey. **(b, c,** and **d)** Cartoon showing the positions of the simulation planes shown in **Fig 6F**.
(TIF)

S1 Microcontroller Code Code for the Arduino microcontroller. (DOCX)

S1 Table ATLIS Control commands. (DOCX)

S2 Table List of prices for all the components of the ATLIS. ^1^Average price from three 3D-printing services located in Uppsala (Sweden); ^2^the shutter disc was fabricated from a piece of plastic; ^3^price of components bought online might vary based on shipping expenses; ^4^price shown is the average of all available bottom plate formats; ^5^the computer power supply used has been discontinued, the price corresponds to a power supply unit that meets the system’s requirements; ^6^prices were paid in Swedish crowns (SEK) and an exchange rate of 1 USD = 8,5 SEK was used; ^7^online sites providing free international shipping to buy the electronic components bought in retail stores.
(DOCX)

S1 Video HEK cell monitoring using the ATLIS. HEK cells grown in a 6-well plate were monitored for 12 h using the ATLIS mounted on a Nikon TMS microscope using the 20X objective together with 10X magnification in the eyepiece. Images were captured every 5 min using a Samsung Galaxy S I9000.
(MOV)

S2 Video Scratch-wound assay. Scratch-wound assay performed with HEK cells grown in a 6-well plate. Cells were monitored for 24 h using the ATLIS mounted on a Nikon TMS microscope using the 20X objective together with 10X magnification in the eyepiece. Images were captured every 5 min using a Samsung Galaxy S I9000.
(MOV)

S3 Video HEK cells grown in microfluidic channels. HEK cells were imaged every 1 min for 4 h with a Samsung Galaxy S I9000 using the ATLIS with the humidifying module in a recirculating-loop configuration. The ATLIS was mounted on a Nikon TMS microscope with 10X magnification in the eyepiece and using a 20X objective.
(MOV)

## References

[1] Gross BC, Erkal JL, Lockwood SY, Chen C, Spence DM. Evaluation of 3D printing and its potential impact on biotechnology and the chemical sciences. Anal Chem. 2014;86(7):3240–53. 10.1021/ac403397r 24432804

[2] Wohlers Associates. Wohlers Report 2016: 3D Printing and Additive Manufacturing State of the Industry Annual Worldwide Progress Report. 2016.

[3] Baden T, Chagas AM, Gage GJ, Marzullo TC, Prieto-Godino LL, Euler T. Open Labware: 3-D printing your own lab equipment. PLoS Biol. 2015;13(3):e1002086 PubMed Central PMCID: PMCPMC4368627. 10.1371/journal.pbio.1002086 25794301 PMC4368627

[4] Wijnen B, Hunt EJ, Anzalone GC, Pearce JM. Open-source syringe pump library. PLoS One. 2014;9(9):e107216 PubMed Central PMCID: PMCPMC4167991. 10.1371/journal.pone.0107216 25229451 PMC4167991

[5] Zhang YS, Ribas J, Nadhman A, Aleman J, Selimovic S, Lesher-Perez SC, et al A cost-effective fluorescence mini-microscope for biomedical applications. Lab Chip. 2015;15(18):3661–9. PubMed Central PMCID: PMCPMC4550514. 10.1039/c5lc00666j 26282117 PMC4550514

[6] Baker M. Cellular imaging: Taking a long, hard look. Nature. 2010;466(7310):1137–40. 10.1038/4661137a 20740018

[7] Frigault MM, Lacoste J, Swift JL, Brown CM. Live-cell microscopy—tips and tools. J Cell Sci. 2009;122(Pt 6):753–67. 10.1242/jcs.033837 19261845

[8] Entschladen F, Drell TLt, Lang K, Masur K, Palm D, Bastian P, et al Analysis methods of human cell migration. Exp Cell Res. 2005;307(2):418–26. 10.1016/j.yexcr.2005.03.029 15950622

[9] Friedl P, Gilmour D. Collective cell migration in morphogenesis, regeneration and cancer. Nat Rev Mol Cell Biol. 2009;10(7):445–57. 10.1038/nrm2720 19546857

[10] Wolfe DB, Qin D, Whitesides GM. Rapid prototyping of microstructures by soft lithography for biotechnology. Methods Mol Biol. 2010;583:81–107. 10.1007/978-1-60327-106-6_3 19763460

