# Supplementary figures and images for: A Modular and Affordable Time-Lapse Imaging and Incubation System Based on 3D-Printed Parts, a Smartphone, and Off-The-Shelf Electronics
Source: PLoS One. 2016 Dec 21;11(12):e0167583. doi: 10.1371/journal.pone.0167583 (PMC5176263; doi:10.1371/journal.pone.0167583)

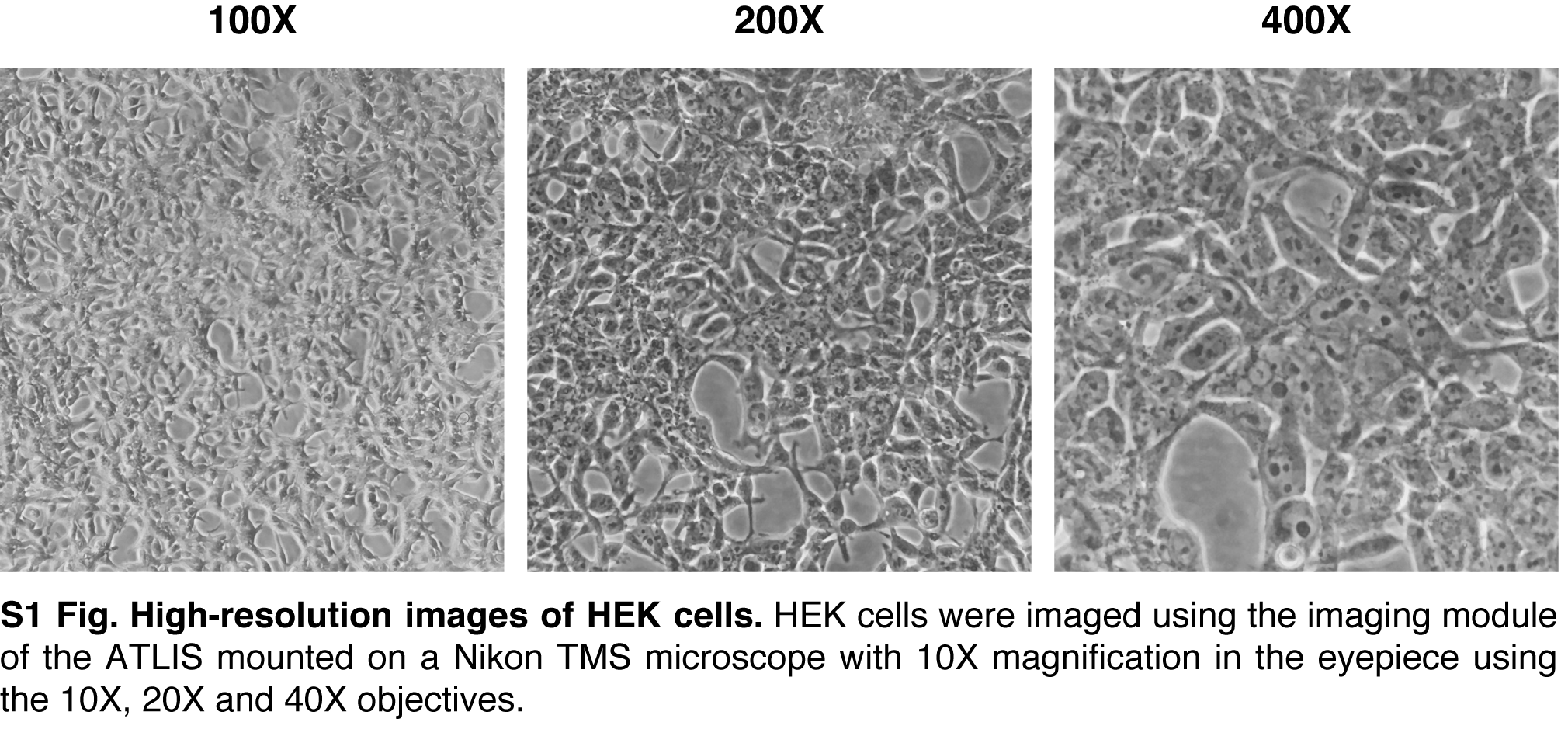

Supplement: S1 Fig — HEK cells were imaged using the imaging module of the ATLIS mounted on a Nikon TMS microscope with 10X magnification in the eyepiece using 10X, 20X and 40X objectives. (TIF) [file pone.0167583.s001.tif]

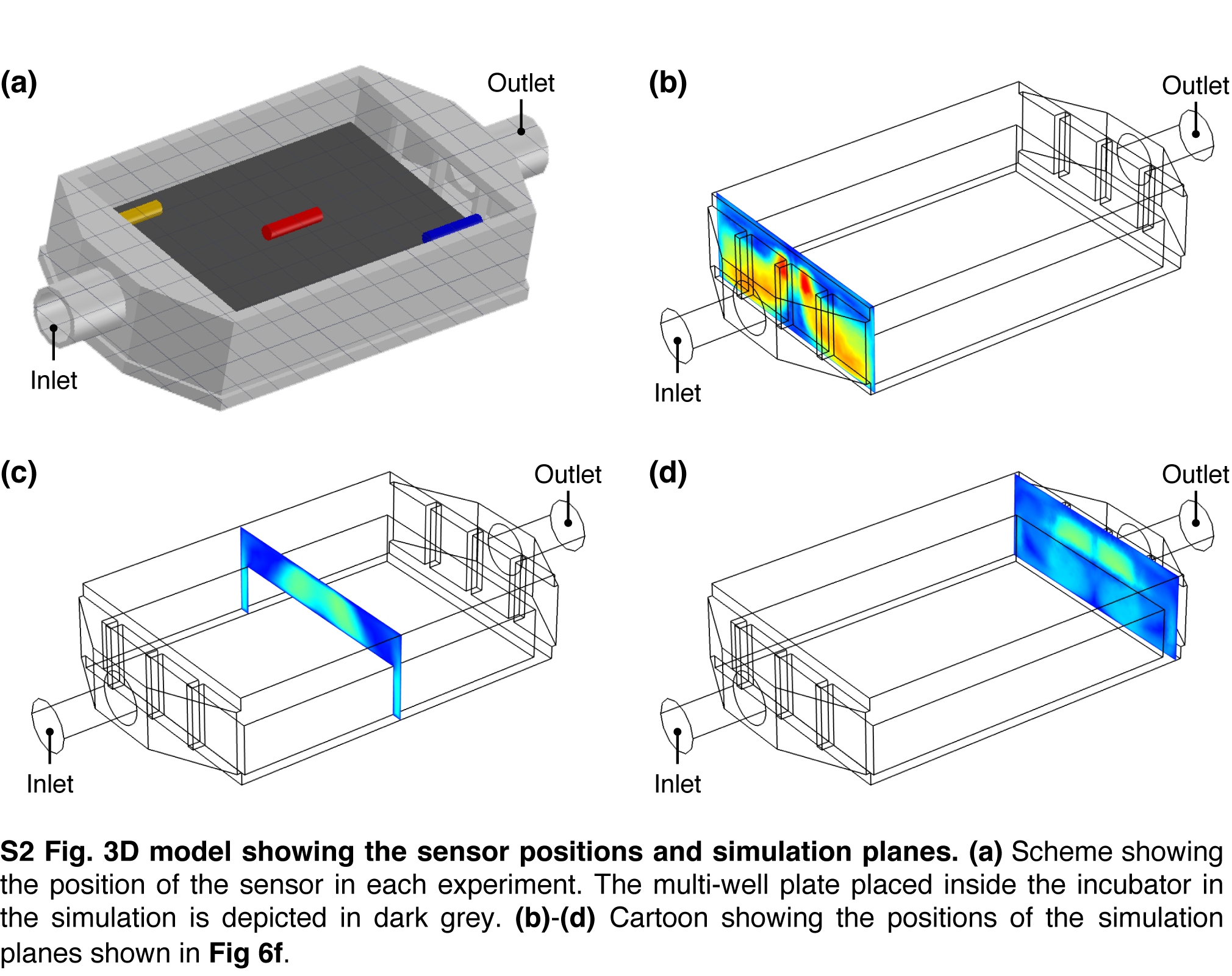

Supplement: S2 Fig — (a) Scheme showing the position of the sensor in each experiment. The multi-well plate placed inside the incubator in the simulation is depicted in dark grey. (b, c, and d) Cartoon showing the positions of the simulation planes shown in Fig 6F. (TIF) [file pone.0167583.s002.tif]
