# Supplementary material for: A Modular and Affordable Time-Lapse Imaging and Incubation System Based on 3D-Printed Parts, a Smartphone, and Off-The-Shelf Electronics
Source: PLoS One. 2016 Dec 21;11(12):e0167583. doi: 10.1371/journal.pone.0167583 (PMC5176263; doi:10.1371/journal.pone.0167583)
Supplement: S1 Microcontroller Code — (DOCX) [file pone.0167583.s003.docx]

**S1 Microcontroller Code**

//include EEPROM

#include <EEPROM.h>

//EEPROM map

//bytes 0 - 7 ControlAddress

//bytes 8 - 11 float P

//bytes 12 - 15 float I

//bytes 16 - 19 float D

//bytes 20 - 23 float targetTemp

//bytes 24 - 31 LimiterAdress

//bytes 32 - 35 float limiterMax

//includes for Servo

#include <Servo.h>

//includes for temperature sensors

#include <OneWire.h>

#include <DallasTemperature.h>

//include for BT module

#include <SoftwareSerial.h>

//include for PID-library

#include <PID_v1.h> //http://playground.arduino.cc/Code/PIDLibrary, https://github.com/br3ttb/Arduino-PID-Library

//include for parsing serial command

#include <SerialCommand.h> //https://github.com/scogswell/ArduinoSerialCommand/

// Data wire is plugged into port 2 on the Arduino

#define ONE_WIRE_BUS 2

//Output pin for heat power

#define outputPin 3

//Temperature resolution

#define TEMPRES 12

//Minimal duration between serial send (ms)

long int serialWait=10000;

//Setup software serial

SoftwareSerial SWSerial=SoftwareSerial(4,5); // RX, TX

//Set up Serial command

SerialCommand swCmd(SWSerial);

// Setup a oneWire instance to communicate with any OneWire devices (not just Maxim/Dallas temperature ICs)

OneWire oneWire(ONE_WIRE_BUS);

// Pass our oneWire reference to Dallas Temperature.

DallasTemperature sensors(&oneWire);

// arrays to hold device address

DeviceAddress Thermometer[5];

//Address of the controling themometer

byte ControlAddress[8];

int ControlDevice=-1;

//Adress of the temperature limiting senros thormometer

byte LimiterAddress[8];

int LimiterDevice=-1;

// Variable to hold number of devices found

int numDev;

//Set up servo

Servo Shutter;

#define SERVOPIN 10

//Vars for temp

double targetTemp;

double currentTemp;

double Temp[5];

double output = 0;

//Var for temp limiter sensor

double limiterMax=50;

double limiterTemp;

#define LOWLIMIT 0.0 //lowest acceptable value to limiter sensor

#define HIGHLIMIT 100.0 //highest acceptable limit to limiter sensor

//Vars for PID

double Kp;

double Ki;

double Kd;

//Var for Shutter position

int ShutterPos=0;

//Var for timings

unsigned long conversionStart=0; //time of last tempoerature conversion start

unsigned long heatPulseStart=0; // time of last heatpulse start

unsigned long heatDutyOff=0; //time when heat duty cycle ends and heater shuts off

int skipSerialPrint=1; //print temp data ony every Nth temperature conversion

int currentPrintSkip=0; //var to hold current skip

unsigned long servoStart=0; //var to hold time of servo start

//var to know if conversion happened this cycle

boolean converted=false;

//var to know if servo was enabled this move

boolean servoMoved=true;

//Time settings

#define CONVTIME 750 //time of conversion in ms

#define TEMPCYCLE 1000 //time to next temperature reading

#define HEATPULSE 2000 //time of heat pulse cycle in ms

#define SERVOTIME 1000 //time to activate servo for a move

//Specify the links and initial tuning parameters

PID myPID(&currentTemp, &output, &targetTemp, Kp, Ki, Kd, DIRECT);

void setup()

{

//Set ouputpin and LED pin to ouput

pinMode(outputPin, OUTPUT); //output pin

pinMode(13,OUTPUT); //LED pin

//initialise serial over USB

Serial.begin(9600);

//Initialise software serial

SWSerial.begin(9600);

//Set up serial commands for software serial

swCmd.addCommand("T",Tcmd); // set Target Temp

swCmd.addCommand("S",Scmd); // set shutter position

swCmd.addCommand("P",Pcmd); // set P in PID

swCmd.addCommand("I",Icmd); // set I in PID

swCmd.addCommand("D",Dcmd); // set D in PID

swCmd.addCommand("R",Rcmd); // request data

swCmd.addCommand("W",Wcmd); // set wait between serial data send

swCmd.addCommand("C",Ccmd); // set controlling thermometer

swCmd.addCommand("L",Lcmd); // set limiter thermometer

swCmd.addCommand("M",Mcmd); // set limiter max

swCmd.addDefaultHandler(unrecognized);

//Read from EEPROM

EEPROM.get(0, ControlAddress);

EEPROM.get(8, Kp);

EEPROM.get(12, Ki);

EEPROM.get(16, Kd);

EEPROM.get(20, targetTemp);

EEPROM.get(24, LimiterAddress);

EEPROM.get(32, limiterMax);

SWprint("Control Address from EEPROM: ");

printAddress(ControlAddress);

SWprintln("");

SWprint("Limiter Address from EEPROM: ");

printAddress(LimiterAddress);

SWprintln("");

SWprintln("Locating devices.");

//initialise the temp sensors on onewWire bus

sensors.begin();

// print number of sensors

numDev=sensors.getDeviceCount();

SWprint("Found ");

SWprinti(numDev);

SWprintln(" devices");

//assign temp sensor address and print it

for (int i=0;i<numDev;i++) {

if (!sensors.getAddress(Thermometer[i], i)) {

SWprint("Error: device ");

SWprinti(i);

SWprintln("");

delay(1000);

} else {

SWprint("Device ");

SWprinti(i);

SWprint(": ");

printAddress(Thermometer[i]);

SWprintln("");

sensors.setResolution(Thermometer[i], TEMPRES);

delay(1000);

//Check if this thermometer is the controlling one

if (ByteArrayCompare(Thermometer[i],ControlAddress,8)) {ControlDevice=i;}

//Check if this thermometer is the limiter

if (ByteArrayCompare(Thermometer[i],LimiterAddress,8)) {LimiterDevice=i;}

}

}

if (ControlDevice>-1) {

SWprint("Control themometer is: ");

SWprinti(ControlDevice);

SWprintln("");

} else {

SWprintln("No control thermometer! Set with C.");

}

if (LimiterDevice>-1) {

SWprint("Limiter themometer is: ");

SWprinti(LimiterDevice);

SWprintln("");

} else {

SWprintln("No limiter thermometer! Set with L.");

}

//set no wait for conversion, let loop run instead

sensors.setWaitForConversion(false);

//initialise PID library

myPID.SetMode(AUTOMATIC);

myPID.SetOutputLimits(0.0,100.0);

SWprintPID();

myPID.SetTunings(Kp, Ki, Kd);

delay(1000);

}

void loop() {

// Request temperature from sensors

if (millis()>conversionStart+TEMPCYCLE) {

sensors.requestTemperatures();

conversionStart=millis();

converted = false;

}

//Read temp if time is right

if (millis()>conversionStart+CONVTIME && converted==false) {

converted=true;

for (int i=0;i<numDev;i++) {

//Read

double oldTemp = Temp[i];

Temp[i] = sensors.getTempC(Thermometer[i]);

//Debounce

//if (Temp[i] == -127.0) {Temp[i] = oldTemp;} //removed to check interference

//if (Temp[i] == 0.0) {Temp[i] = oldTemp;}

//if (Temp[i] == 85.0) {Temp[i] = oldTemp;}

if (i==ControlDevice) {currentTemp=Temp[i];}

if (i==LimiterDevice) {limiterTemp=Temp[i];}

}

//PID recalculate with new temps

myPID.Compute();

//increase serial skip variable

currentPrintSkip++;

//Draw tempdata if skipped enough

if (currentPrintSkip >= skipSerialPrint && skipSerialPrint>0) {

if (servoMoved==false) {Shutter.detach();}

SWprintTemp();

if (servoMoved==false) {Shutter.attach(SERVOPIN);}

currentPrintSkip=0;

}

}

//Output heatpulse

if (millis()>heatPulseStart+HEATPULSE && limiterTemp <= limiterMax && limiterTemp <= HIGHLIMIT && limiterTemp >= LOWLIMIT) {

digitalWrite(outputPin, HIGH);

digitalWrite(13,HIGH);

heatPulseStart=millis();

heatDutyOff=map(int(output),0,100,heatPulseStart,heatPulseStart+HEATPULSE);

}

if (millis()>heatDutyOff) {

digitalWrite(outputPin,LOW);

digitalWrite(13,LOW);

}

//Read serial command

swCmd.readSerial();

//Servo control

if (millis()>servoStart+SERVOTIME && servoMoved==false) {

Shutter.detach();

servoMoved=true;

}

}

void SWprintTemp() {

SWprint("Ti:");

SWprintul(millis());

SWprint(" Ta:");

SWprintd(targetTemp);

SWprint(" O:");

SWprinti(int(output));

for (int i=0;i<numDev;i++) {

SWprint(" ");

SWprinti(i);

SWprint(":");

SWprintd(Temp[i]);

}

SWprintln("");

}

void SWprintFull() {

SWprint("Have ");

SWprinti(numDev);

SWprintln(" devices");

//assign temp sensor address and print it

for (int i=0;i<numDev;i++) {

SWprint("Device ");

SWprinti(i);

SWprint(": ");

printAddress(Thermometer[i]);

SWprintln("");

}

SWprint("Control themometer is: ");

SWprinti(ControlDevice);

SWprint(": ");

printAddress(ControlAddress);

SWprintln("");

SWprint("Limiter themometer is: ");

SWprinti(LimiterDevice);

SWprint(": ");

printAddress(LimiterAddress);

SWprintln("");

SWprint("Limiter max temp is: ");

SWprintd(limiterMax);

SWprintln("");

SWprintPID();

SWprint("Shutter Position: ");

SWprinti(ShutterPos);

SWprintln("");

SWprint("Serial wait: ");

SWprintl(skipSerialPrint);

SWprintln("");

}

//Print to both Serial and SWSerial

void SWprint(char *arg) { //String

Serial.print(arg);

SWSerial.print(arg);

}

void SWprintln(char *arg) { // String + end of line

Serial.println(arg);

SWSerial.println(arg);

}

void SWprinti(int i) { //int

Serial.print(i);

SWSerial.print(i);

}

void SWprinth(int h) { //hex

Serial.print(h, HEX);

SWSerial.print(h, HEX);

}

void SWprintd(double d) { //double

Serial.print(d);

SWSerial.print(d);

}

void SWprintul(unsigned long ul) { //unsigned long

Serial.print(ul);

SWSerial.print(ul);

}

void SWprintl(long l) { //long

Serial.print(l);

SWSerial.print(l);

}

void SWprintPID() {

SWprint("PID set to: ");

SWprintd(Kp);

SWprint(" ");

SWprintd(Ki);

SWprint(" ");

SWprintd(Kd);

SWprintln("");

}

// function to print a device address

void printAddress(DeviceAddress deviceAddress)

{

for (uint8_t i = 0; i < 8; i++)

{

if (deviceAddress[i] < 16) {

SWprint("0");

}

SWprinth(deviceAddress[i]);

}

}

boolean ByteArrayCompare(byte a[], byte b[], int array_size)

{

for (int i = 0; i < array_size; ++i)

if (a[i] != b[i])

return(false);

return(true);

}

//Commands received

void Tcmd() {

char *arg;

arg = swCmd.next();

if (arg != NULL) {

targetTemp=atof(arg);

SWprint("Target set to: ");

SWprintd(targetTemp);

SWprintln("");

EEPROM.put(20,targetTemp);

} else {

SWprintln("T: No data.");

}

}

void Scmd() {

char *arg;

arg = swCmd.next();

if (arg != NULL) {

ShutterPos=atoi(arg);

Shutter.write(ShutterPos);

Shutter.attach(SERVOPIN);

servoStart=millis();

servoMoved=false;

} else {

SWprintln("S: No data.");

}

}

void Pcmd() {

char *arg;

arg = swCmd.next();

if (arg != NULL) {

Kp=atof(arg);

SWprintPID();

myPID.SetTunings(Kp, Ki, Kd);

EEPROM.put(8,Kp);

} else {

SWprintln("P: No data.");

}

}

void Icmd() {

char *arg;

arg = swCmd.next();

if (arg != NULL) {

Ki=atof(arg);

SWprintPID();

myPID.SetTunings(Kp, Ki, Kd);

EEPROM.put(12,Ki);

} else {

SWprintln("I: No data.");

}

}

void Dcmd() {

char *arg;

arg = swCmd.next();

if (arg != NULL) {

Kd=atof(arg);

SWprintPID();

myPID.SetTunings(Kp, Ki, Kd);

EEPROM.put(16,Kd);

} else {

SWprintln("D: No data.");

}

}

void Wcmd() {

char *arg;

arg = swCmd.next();

if (arg != NULL) {

skipSerialPrint=atoi(arg);

} else {

SWprintln("W: No data.");

}

}

void Rcmd() {

char *arg;

arg=swCmd.next();

int i;

if (arg != NULL) {

i=atoi(arg);

switch(i) {

case 1:

SWprintTemp();

break;

case 2:

SWprintFull();

break;

default:

SWprintln("R: Unrecognised.");

break;

}

} else {

SWprintln("R: No data.");

}

}

void Ccmd() {

char *arg;

arg = swCmd.next();

if (arg != NULL) {

ControlDevice=atoi(arg);

if (ControlDevice>numDev-1 || ControlDevice < 0) {

ControlDevice=-1;

SWprintln("C: Device does not exist.");

} else {

for (int i=0;i<8;i++) {

ControlAddress[i] = Thermometer[ControlDevice][i];

}

EEPROM.put(0,Thermometer[ControlDevice]);

}

} else {

SWprintln("C: No data.");

}

}

void Lcmd() {

char *arg;

arg = swCmd.next();

if (arg != NULL) {

LimiterDevice=atoi(arg);

if (LimiterDevice>numDev-1 || LimiterDevice < 0) {

LimiterDevice=-1;

SWprintln("L: Device does not exist.");

} else {

for (int i=0;i<8;i++) {

LimiterAddress[i] = Thermometer[LimiterDevice][i];

}

EEPROM.put(24,Thermometer[LimiterDevice]);

}

} else {

SWprintln("L: No data.");

}

}

void Mcmd() {

char *arg;

arg = swCmd.next();

if (arg != NULL) {

limiterMax=atof(arg);

SWprint("Limiter temperature set to: ");

SWprintd(limiterMax);

SWprintln("");

EEPROM.put(32,limiterMax);

} else {

SWprintln("M: No data.");

}

}

void unrecognized() {

SWprintln("Command Unrecognised.");

}
