# Supplementary material for: A Modular and Affordable Time-Lapse Imaging and Incubation System Based on 3D-Printed Parts, a Smartphone, and Off-The-Shelf Electronics
Source: PLoS One. 2016 Dec 21;11(12):e0167583. doi: 10.1371/journal.pone.0167583 (PMC5176263; doi:10.1371/journal.pone.0167583)
Supplement: S1 Table — (DOCX) [file pone.0167583.s004.docx]

**S1 Table. ATLIS Control commands**

| **COMMAND** | **FUNCTION** |
| --- | --- |
| T | Set target temperature |
| C | Set controlling temperature sensor |
| L | Set limiting temperature sensor |
| M | Set heating unit maximum temperature |
| P | Set K_P_ value |
| I | Set K_I_ value |
| D | Set K_D_ value |
| W | Set time between temperature recordings |
| S | Set shutter position |
| R 1 | Request a temperature read |
| R 2 | Request full system settings |
