# Supplementary material for: A Modular and Affordable Time-Lapse Imaging and Incubation System Based on 3D-Printed Parts, a Smartphone, and Off-The-Shelf Electronics
Source: PLoS One. 2016 Dec 21;11(12):e0167583. doi: 10.1371/journal.pone.0167583 (PMC5176263; doi:10.1371/journal.pone.0167583)
Supplement: S2 Table — 1Average price from three 3D-printing services located in Uppsala (Sweden); 2the shutter disc was fabricated from a piece of plastic; 3price of components bought online might vary based on shipping expenses; 4price shown is the average of all available bottom plate formats; 5the computer power supply used has been discontinued, the price corresponds to a power supply unit that meets the system’s requirements; 6prices were paid in Swedish crowns (SEK) and an exchange rate of 1 USD = 8,5 SEK was used; 7online sites providing free international shipping to buy the electronic components bought in retail stores. (DOCX) [file pone.0167583.s005.docx]

**S2 Table. List of prices for all the components of the ATLIS.**

| **Component** | **Quantity** | **Price (USD)^6^** | **Retailer** | **Online alternative^7^** |
| --- | --- | --- | --- | --- |
| **(A) Imaging module** |  |  |  |  |
| Smartphone holder^1^ | 1 | 21.46 | - | - |
| Holders^1^ | 3 | 8.51 | - | - |
| 3D-printed nuts^1^ | 5 | 7.62 | - | - |
| Servomotor | 1 | 11.75 | http://bit.ly/servo-atlis | http://bit.ly/alt-servo |
| Shutter disc^2^ | 1 | 0.12 | - | - |
| Holder | 1 | 7.33 | - | - |
| 1 mm thick rubber spacers | 2 | 0.16 | http://bit.ly/rubber-atlis | - |
| M4 screws (40 mm long) | 3 | 0.17 | - | - |
| M4 threaded rod (8 cm) | 2 | 0.19 | - | - |
| M4 washers | 8 | 0.16 | - | - |
| M4 nuts | 7 | 0.11 | - | - |
|  | **Total** | **57.57** |  |  |
| **B) Heating unit** |  |  |  |  |
| Heat resistant hose (10 cm) | 1 | 0.39 | - | - |
| Case | 1 | 19.23 | - | - |
| 60 mm 12 V fan | 1 | 8.22 | http://bit.ly/fan-atlis | http://bit.ly/alt-fan |
| 4.5 screws (50 mm long) | 4 | 0.09 | - | - |
| Temperature sensor^3^ | 1 | 0.94 | http://bit.ly/tsensor-atlis | - |
| 40 W heating element (12 V)^3^ | 1 | 8.24 | http://bit.ly/heating-atlis | - |
| 1mm wire (50 cm) | 1 | 0.07 | - | - |
| 20 mm x 20 mm aluminum (5 cm) | 2 | 1.40 | - | - |
| M4 screws (50 mm long) | 2 | 0.09 | - | - |
| M4 washers | 2 | 0.08 | - | - |
| M4 nuts | 2 | 0.03 | - | - |
|  | **Total** | **38.77** |  |  |
| **C) Incubation module** |  |  |  |  |
| Lid^1^ | 1 | 15.76 | - | - |
| 2 mm thick plexiglas (8x12 cm) | 1 | 0.50 | http://bit.ly/plexiglas-atlis | - |
| Body^1^ | 1 | 28.51 | - | - |
| Bottom^1,4^ | 1 | 17.70 | - | - |
| Temperature sensor^3^ | 1 | 0.94 | http://bit.ly/tsensor-atlis | - |
|  | **Total** | **63.41** |  |  |
| **D) Control unit** |  |  |  |  |
| Printed circuit board (5x7 cm)^3^ | 1 | 0.12 | http://bit.ly/pcb-atlis | - |
| Arduino Nano 3.0 ATmega328 | 1 | 41.06 | http://bit.ly/arduino-atlis | http://bit.ly/alt-arduino |
| Bluetooth module | 1 | 15.28 | http://bit.ly/btmodule-atlis | http://bit.ly/alt-bt |
| Resistors | 1 | 0.29 | http://bit.ly/res-atlis | http://bit.ly/alt-resistors |
| Power transistor^3^ | 1 | 0.25 | http://bit.ly/pwrtrans-atlis | - |
| Voltage regulator^3^ | 1 | 0.11 | http://bit.ly/voltreg-atlis | - |
|  | **Total** | **57.12** |  |  |
| **E) Power supply unit** |  |  |  |  |
| Power supply unit^5^ | 1 | 36.35 | http://bit.ly/psu-atlis | http://bit.ly/alt-psu |
|  | **Total** | **36.35** |  |  |
| **F) Humidifying module** |  |  |  |  |
| Air pump (Sera air 110 plus) | 1 | 21.06 | http://bit.ly/pump-atlis |  |
| Airstone | 1 | 2.34 |  |  |
|  | **Total** | **23.40** |  |  |
|  |  |  |  |  |
| **ATLIS Total** |  | **276.63** |  |  |

^1^Average price from three 3D-printing services located in Uppsala (Sweden); ^2^the shutter disc was fabricated from a piece of plastic; ^3^price of components bought online might vary based on shipping expenses; ^4^price shown is the average of all available bottom plate formats; ^5^the computer power supply used has been discontinued, the price corresponds to a power supply unit that meets the system’s requirements; ^6^prices were paid in Swedish crowns (SEK) and an exchange rate of 1 USD = 8,5 SEK was used; ^7^online sites providing free international shipping to buy the electronic components bought in retail stores.
